# Transcription activation mechanism of a non-canonical bacterial DNA damage response pathway

**DOI:** 10.21203/rs.3.rs-7152246/v1

**Published:** 2025-08-05

**Authors:** Rajiv R. Singh, Amani Chinni, Emily Cannistraci, Raul Salinas, Kevin Gozzi, Maria A. Schumacher

**Affiliations:** 1 Department of Biochemistry, 307 Research Dr., Box 3711, Duke University Medical Center, Durham, NC 27710, USA.; 2 Rowland Institute at Harvard, 52 Oxford St, Harvard University, Cambridge, MA 02138, USA.

## Abstract

DNA damage repair mechanisms are vital for bacterial survival. Recent studies revealed a non-canonical DNA damage response in *Caulobacter crescentus* activated by a WYL-domain transcription factor, DriD. DriD binds ssDNA, produced upon DNA damage, within its WYL-domain, and drives expression at multiple promoters. The mechanism behind DriD-mediated transcription activation is, however, unknown. Here we describe cryo-EM structures of DriD-ssDNA bound to RNAP-holoenzyme and three promoters. DriD contains N-terminal DNA-binding domains (DNABDs) connected to WYL-signaling domains by a linker-3-helix-bundle (3HB) module. The three structures reveal a conserved activation mechanism whereby DriD’s 3HBs bind RNAP α-CTD and ß domains, anchoring RNAP on nonoptimal promoters. The 3HBs form autoinhibitory contacts with DNABDs in apo DriD and therefore acts as a ssDNA-driven trigger domain, switching between DNABD-bound apo and RNAP-bound states upon ssDNA-mediated activation. Thus, the structures reveal a unique transcription activation mechanism, likely conserved among the large family of homodimeric WYL-activators.

## Introduction

The maintenance of genomic integrity during DNA damage is essential for cell survival^1–4^. Most bacteria employ a canonical DNA damage repair pathway called the SOS response^2–6^. The trigger for the SOS response is single stranded DNA (ssDNA), which accumulates during DNA damage from replication fork stalling and damage such as double stranded DNA breaks^7–11^. ssDNA binds to RecA, which functions as a coprotease to enable self-cleavage of the SOS master transcription repressor, LexA. Once cleaved, LexA can no longer bind its operator DNA sites, allowing transcription of the SOS regulon, including cell cycle checkpoint proteins^2–25^. Cell cycle inhibitors play central roles in this process as, by halting cell division, they allow time for DNA repair, thus preventing the propagation of damaged chromosomes. The SOS DNA damage response was initially thought to be the primary DNA repair pathway in bacteria. However, recent studies have uncovered noncanonical DNA damage responses^26–36^. One such pathway was characterized in *Caulobacter crescentus*^36^. This pathway activates the production of a cell division regulator called DidA, which binds to the late arriving divisome protein, FtsN, to pause cell division^36^. Subsequent work showed that DidA expression was activated by a 327-residue transcription factor, originally identified as a member of the DeoR family of regulators. Hence, the protein was called DriD for DeoR inducer of *d**idA*^36^.

Although containing an N-terminal winged helix-turn-helix (wHTH) domain as in other DeoR family members, DriD’s sequence placed it in the recently characterized WYL family of proteins. These proteins are predominantly found in bacteria, with the WYL designation arising from the presence of a Trp-Tyr-Leu motif in their sequences^36–47^. Like DriD, the majority of these proteins contain winged wHTH motifs and function as transcription factors (TFs)^41,43^. Studies on DriD showed it binds in a sequence-specific manner to a 20 bp pseudo palindromic target DNA site^38^. These analyses showed that DriD functions as a transcriptional activator, not a repressor, as is LexA. In addition to *didA*, subsequent studies revealed DriD target DNA binding sites in promoters regulating the *bapE, recJ* and *dnaG* genes^37–39^. *bapE* encodes an endonuclease that leads to an apoptosis like response during severe DNA damage, *recJ* encodes an exonuclease involved in DNA repair and *dnaG* encodes a primase that has been shown to be involved in DNA repair pathways^48–50^. In addition, a DriD binding site was also found upstream of the *driD* gene itself^37^. With the exception of *driD*, these genes were all shown to be activated by DriD in a DNA-damage induced manner in conjunction with the RNAP-σ73 holoenzyme^37^, where *C. crescentus* σ73 is the homolog of the housekeeping σ70 subunit in other bacteria. The bacterial RNAP core, which is composed of the catalytic β and β’ subunits, two copies of the α subunit and ω, shows overall structural similarity to eukaryotic and archaeal RNAPs. However, promoter recognition in bacteria is mediated by σ factors. All bacteria encode the housekeeping σ70, which recognizes specific promoter elements 6 nucleotides in length, located −35 and −10 bps upstream of the transcription state site^37^. Interestingly, the DriD DNA-binding sites in its regulated promoters appear to be located in different positions^37^.

Structures capturing ssDNA bound to the DriD WYL-motifs suggested that ssDNA may act as an allosteric regulator^37^. This was confirmed by biochemical and cell biology studies, which showed that ssDNA binding to DriD stimulated its interaction with target DNA and led to activation of DriD regulated promoters^37–38^. A crystal structure of DriD in complex with ssDNA and target DNA showed that each subunit of the homodimeric DriD is comprised of an N-terminal wHTH DNA binding domain linked to a core region containing a three-helix-bundle (3HB), WYL and (WCX) dimerization domains^38^. The structure revealed that when in complex with ssDNA, DriD recognizes its target DNA by binding in an unusual asymmetrical manner and imparting an overall ~30° bend to its DNA site. DriD recognizes its target DNA site with high specificity primarily through the formation of bidentate hydrogen bonds from arginine residues to the conserved guanine bases in each half site of the DNA consensus, ATACGAC(X)_7_GTCGTAT (where the underlined nucleotides are the contacted guanines). Indeed, experiments showed that these guanines are essential for high affinity target DNA binding by DriD^38^. Recent HDX-MS and biochemical analyses revealed that allosterism is imparted by ssDNA binding to the DriD WYL-domains via conformational changes that are transduced to the 3HBs. The result is a release of autoinhibitory interactions between the DriD DNABDs and 3HBs that are formed in the apo state, allowing for target DNA binding^51^.

Accumulating data indicates that DriD is representative of a large family of homodimeric WYL-activators^41^. However, while studies have uncovered ssDNA as the allosteric effector of DriD and its mode of target DNA recognition, it is unclear how DriD or other homodimeric WYL-activators interface with RNAP to activate transcription. Indeed, DriD harbors a structure that is distinct from previously solved bacterial transcription activators^52^ and there is currently limited structural information on the *C. crescentus* RNAP machinery^53^. Thus, to elucidate the transcription activation mechanism behind the master regulator, DriD, of this SOS-independent DNA response pathway we employed cryo-electron microscopy (cryo-EM). We obtained structures of DriD-ssDNA-RNAP-σ73 complexes bound to the *driD* and *didA* promoters, which contain predicted DriD target sites located proximal to the −10 and −35 elements, respectively. We also solved a structure of DriD-ssDNA-RNAP-σ73 in complex with the *bapE* promoter, which contains unidentifiable −35/−10 DNA elements (Supplementary Figure 1). Strikingly, the structures all reveal the same assembly of DriD on a DNA site that overlaps the −35 promoter element. In the structures, DriD makes contacts to the RNAP β subunit and one of the αCTDs, anchoring the RNAP holoenzyme onto promoters with non-optimal −35 and −10 promoter elements. The assembly on the *driD* promoter was explained by our finding that this promoter is bidirectional, with one direction encoding *driD* and the other, a two-protein operon composed of *CCNA_03891/CCNA_01149*. Cellular studies confirmed that DriD activates the *CCNA_03891/CCNA_01149* promoter and not the *driD* gene. Collectively, these studies have uncovered a new operon regulated by DriD and show that it employs a conserved mode of transcription activation that is distinct from those employed by previously characterized transcription activators.

## Results

### *C. crescentus* DriD-ssDNA-RNAP-σ73 in complex with *driD* promoter DNA

**Figure 1.**
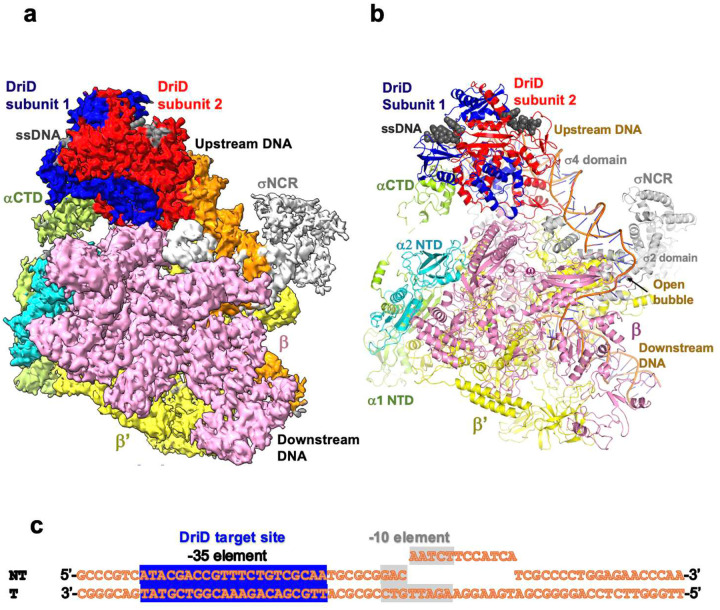
Overall cryo-EM structure of the *C. crescentus* DriD-ssDNA-RNAP-σ73-*CCNA_03891/CCNA_01149* promoter complex. **(a)** Cryo-EM density map for the *C. crescentus* DriD-ssDNA-RNAP-σ73-*CCNA_03891/CCNA_01149* promoter complex with DriD subunits, and regions contacting DriD, the αCTD and β labeled. (**b**) Ribbon diagram of the *C. crescentus* DriD-ssDNA-RNAP-σ73-*CCNA_03891/CCNA_01149* promoter complex shown in the same orientation as (a) with DriD subunits labeled. RNAP subunits are labeled as are the domains of the σ73 subunit. The upstream and downstream DNA are labeled as is the open bubble formed at the active site near σ2. (**c**) Sequence of the promoter bound to the *C. crescentus* DriD-ssDNA-RNAP-σ73-*CCNA_03891/CCNA_01149* promoter complex. The DNA shown includes the region that was visible in the structure. The DriD target DNA site is highlighted in blue and the −10 element in grey.

Previous studies revealed that DriD activates transcription from multiple promoters^37^. To elucidate the mechanism(s), we generated *C. crescentus* DriD-ssDNA-RNAP-σ73 holoenzyme complexes bound to three promoters shown to contain DriD binding sites, *didA, driD* and *bapE* (Supplementary Figure 1; Supplementary Table 1; Methods). The DriD-ssDNA-RNAPσ73-*driD* promoter cryo-EM structure was solved first to a global resolution of 3.7 Å (Figure 1a; Supplementary Movie 1; Supplementary Figure 2)^54–57^. Density was evident for DriD-ssDNA and the β, β’, ω and the α subunit NTDs of the RNAP core and most residues of σ73 except N-terminal region 1.1 (Methods). As observed in other RNAP complexes, the large β and β’ subunits combine to form a claw-like structure with the catalytic center located in the cleft between the two subunits. The ω subunit primarily interacts with β’. The two identical α subunits homodimerize via their N-terminal domains (NTDs) and the αNTD dimer binds and facilitates assembly of the β/β’ subunits to form the active site (Figure 1a–b). The DNA promoter site used to obtain the structure did not contain a preformed bubble near the −10 element (i.e. did not contain non-complementary DNA). But the promoter density reveals that the −10 DNA element is melted and thus adopts an open promoter, activated state (Figure 1a–b).

The DriD dimer bound at the promoter adopts the same overall structure as observed in crystal structures, whereby each DriD subunit of the dimer consists of five regions, an N-terminal wHTH DNA binding domain (residues 1–70), linker region (residues 71–78), three helix bundle (3HB) domain (residues 79–133), WYL-domain (residues 134–245) and the WYL C-terminal extension, dimer domain (WCX) (residues 246–327) (Supplementary Figure 3)^38^; the Cα atoms of the DriD dimer crystal structure overlay with a root mean square deviation (rmsd) of 1.5 Å onto the DriD dimer from the cryo-EM model (Supplementary Figure 4; Figure 1a–b). As in the crystal structure, the DriD 3HB-WYL-WCX core domains are arranged at a sharp angle relative to the DNA binding domains (see Figure 1a–b; Supplementary Figure 3). Notably, the DriD binding site in this promoter was predicted to be proximal to the −10 hexamer element (Supplementary Figure 1). However, DriD is instead bound to a DNA site located upstream in the promoter, distant from the −10 motif.

### The *driD* promoter is bidirectional and DriD activates the divergent operon

**Figure 2.**
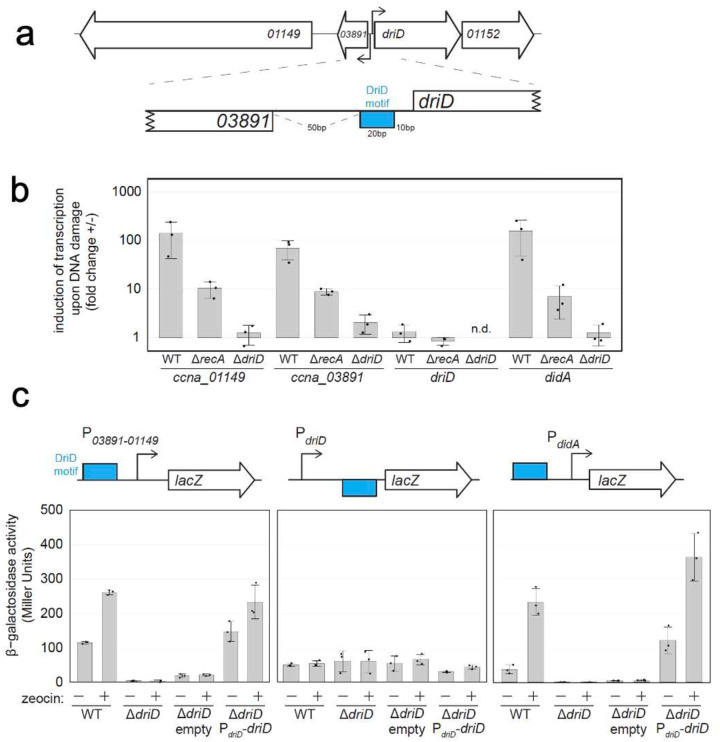
The *driD/CCNA_03891/CCNA_01149* promoter is bidirectional with DriD activating transcription of *CCNA_03891/CCNA_01149*. (**a**) Organization of the *driD/CCNA_03891/CCNA_01149* bidirectional promoter. A zoom-in of the intergenic region between *CCNA_03891* and *driD* with the DriD-binding motif (colored blue). (**b**) qRT-PCR of expression of *CCNA_01149, CCNA_03891, driD* and *didA* relative to the endogenous control *rpoA* in WT, *rec526* and Δ*driD* cells in response to the DNA damaging agent zeocin. qRT-PCR was performed in biological and technical triplicate. Individual data points with mean and standard deviation indicated. (**c**) β-galactosidase activity assays of the *CCNA_03891/CCNA_01149* and driD, alongside *didA* as a positive control, in different genetic backgrounds (WT, Δ*driD*, Δ*driD* + empty vector, Δ*driD* + P_*driD*_-*driD* complementation) with and without zeocin treatment. DriD activates the *CCNA_03891/CCNA_01149* promoter to a similar degree as the *didA* positive control but no activation is seen of the *driD* promoter.

As noted, in the DriD-ssDNA-RNAP-σ73-*driD* promoter complex, DriD is not bound near the −10 DNA promoter element, which would be expected if it were activating its own gene (Figure 1a–b; Supplementary Figure 1)^37^. Instead, in the structure, the RNAP complex, including the bound DriD, is oriented in the opposite direction to that expected for activation of the *driD* gene and DriD is bound close to where the −35 promoter element would be positioned (Supplementary Figure 1; Figure 1a–c). Closer examination of the promoter structure and surrounding gene organization revealed the presence of a divergent operon from *driD* encoding two proteins (CCNA_03891 and CCNA_01149)^57–59^. The *CCNA_03891* gene codes for a gene of unknown function while *CCNA_01149* encodes a putative TonB-dependent transporter (Figure 2a; Figure 1c).

Consistent with the structure indicating that DriD is not positioned to regulate its own expression, but rather that of the *CCNA_03891/CCNA_01149* operon, previous studies had shown that DriD levels do not change upon DNA damage or during the cell cycle^36^. However, to test this hypothesis, we first employed quantitative real-time PCR (qRT-PCR). We measured changes in transcription of *driD*, *CCNA_03891/CCNA_01149*, and *didA* (a positive control) in various genetic backgrounds in the presence or absence of the DNA damage agent zeocin, which induces DriD-mediated transcription activation^36–37^. *CCNA_03891*, *CCNA_01149* and *didA* each showed strong activation in wild-type (WT) cells, diminished but still inducible in *recA*-null cells (*rec526*, SOS response not inducible), and no activation in Δ*driD* cells, supporting the hypothesis that DriD is not regulating its own promoter but rather that of the *CCNA_03891/CCNA_01149* operon (Figure 2a).

To directly test *driD*-mediated activation of promoters, we constructed reporter strains of the *CCNA_03891/CCNA_01149*, *driD* and *didA* promoters fused to *lacZ*, which were integrated at a distal locus on the genome, with concomitant deletion of *driD* at its native locus. DriD complementation was performed in this background by providing a vector containing either no *driD* (empty) or WT *driD* driven by its native promoter (P_*driD*_-*driD*). The strains were then treated with zeocin to induce DriD-mediated activation, which was measured by β-galactosidase production. The *CCNA_03891/CCNA_01149* and *didA* promoters were inducible in both a WT and *driD* complement background but uninducible in a *driD* deletion (Figure 2c). Furthermore, the *driD* promoter showed consistent activity, regardless of the presence of DriD or zeocin. These experiments showed unequivocally that DriD does not regulate its own expression but significantly activates the transcription of the *CCNA_03891/CCNA_01149* genes, consistent with the cryo-EM data.

### The DriD asymmetric binding mode on DNA is key for RNAP-σ73 contacts

**Figure 3.**
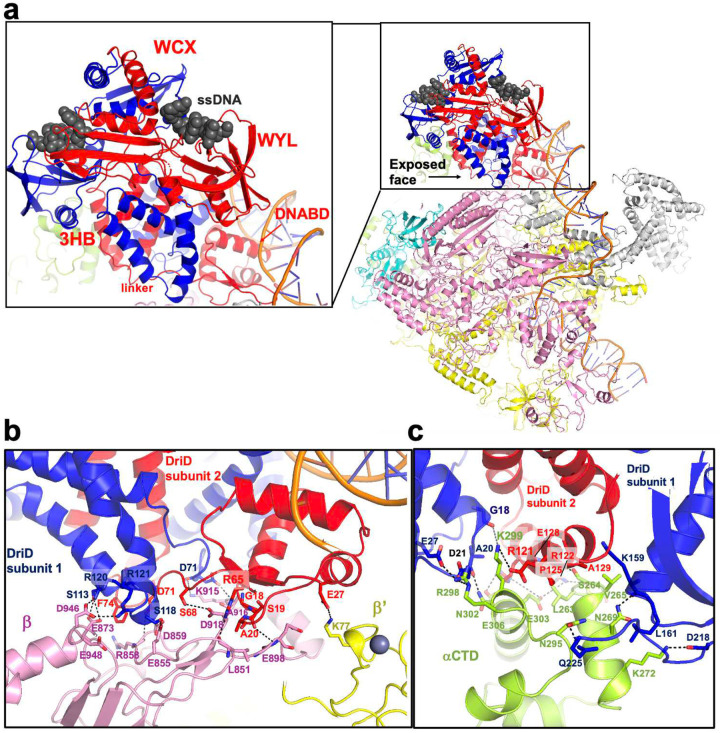
Interactions between RNAP and DriD in the *C. crescentus* DriD-ssDNA-RNAP-σ73-*CCNA_03891/CCNA_01149* promoter complex. **(a)** Ribbon diagram of the complex in Fig. 1b underscoring the asymmetric binding of DriD whereby the 3HB-WYL-WCX core is oriented almost perpendicular to the DNA creating an exposed face on the DriD dimer for RNAP interaction. Shown to the left is a close up of the DriD dimer with each domain labeled. (**b**) Close up of the DriD interactions with the RNAP β subunit (**c**). Close up of the contacts between DriD and the αCTD.

Our transcription assays confirmed that DriD activates transcription of the *CCNA_03891/CCNA_01149* operon (Figure 2b–c). As noted, DriD adopts an unusual, asymmetric orientation on the DNA in the cryo-EM structure. While this mode of binding was observed in all the DriD-ssDNA-target DNA crystal structures, its relevance was unclear in the absence of RNAP^38^. The cryo-EM structure reveals that this conformation optimally positions an exposed face on DriD, composed of the 3HBs and regions of its DNABD, onto the RNAP surface, allowing for multiple interactions with the polymerase complex (Figure 3a; Supplementary Figure 4). The main RNAP regions contacted by DriD are the β subunit and one of the α subunit CTDs, with one contact from DriD to the RNAP β’ subunit, which is from DriD residue Glu27 to β’ residue Lys77 (Figure 3a–c).

In the DriD-β subunit interface, which buries a total of 890 Å^2^ of protein surface from solvent, DriD 3HBs residues 118–121 of one DriD subunit are positioned to interact with residues 855–948 of the β subunit. In particular, DriD 3HB residues Arg120 and Arg121 interact with RNAP β subunit residues Glu873 and Asp946 and make electrostatic interactions with Glu948 and DriD residue Ser118 makes a hydrogen bond with β subunit Glu855. The carbonyl oxygen of residue Asp71 from the linker region, which connects the DNABD to the 3HB, of this DriD subunit also contacts the backbone of β subunit residue Lys915 while the Lys915 amide nitrogen contacts the DriD Asp71 side chain (Figure 3b). Residues from the 3HB, DNABD and linker region of the other subunit of DriD make more extensive contacts with the β subunit (Figure 3b). In these interactions, the side chain of Phe74 from the 3HB of this DriD subunit inserts into a hydrophobic pocket in the β subunit while DriD residue Asp71 contacts backbone atoms of β. Also, from this DriD subunit, the carbonyl of Ser19 interacts with the amide nitrogen of β residue Glu898 and DriD residue Arg65 contacts the β subunit backbone carbonyl oxygen of Leu851 (Figure 3b). Finally, residues Gly18 and Ala20 of this DriD subunit pack tightly against the β subunit such that residues with larger side chains would be predicted to be sterically prohibitive to the DriD-β interaction (Figure 3b).

Comparison of the DriD bound RNAP complex with RNAP structures in open and closed promoter states, which are available for the *Escherichia coli* RNAP^60–62^, show that the region of the β subunit in complex with DriD does not undergo significant conformational changes upon open promoter complex formation (i.e. The DriD bound β subunit in the complex superimposes with the β subunit from *E. coli* RNAP structures with open (7DY6) and closed promoter states (8TOM) with rmsds of 1.5 Å and 1.7 Å for 1340 similar Cα atoms). Hence, the contacts from DriD to the RNAP β subunit do not induce or favor structural changes in β and instead appear to function in stabilizing the RNAP holoenzyme onto the promoter.

Contacts to the RNAP αCTD are provided by DriD residues located on the other face of the dimer (Figure 1a, 3c). In this interface, which buries 920 Å^2^ of the DriD and αCTD surfaces from solvent, helix 3 from the DriD 3HB forms a scaffold that the αCTD slots into (Figure 3c). DriD 3HB residue Pro125 causes a kink in the helix that facilitates the close docking of the helix onto the αCTD surface and the small size of the proline side chain fits within the shallow hydrophobic patch located on the interlacing face of the αCTD. In addition to Pro125, residues Arg121 and Arg122 from this DriD helix are positioned to contact αCTD acidic residues Glu303 and Glu306, which helps lock the DriD helix onto the αCTD (Figure 3c). Asp218 and Gln225 from the WYL domain of the other DriD subunit interact with αCTD residues Lys272 and Asn295, providing further anchoring contacts.

Interestingly, DriD residues Gly18 and Ala20 from the DriD subunit that does not interact with the RNAP β subunit are also involved in this interface. Here, as in the contacts with β, the small size of these residues appears to be important in allowing the formation of this close interface. Notably, the DriD-bound αCTD is 15 Å from the DNA and thus does not bind promoter DNA. In fact, αCTD residues Arg298 and Lys299, which have been shown to interact with AT-rich DNA elements called upstream elements (UP) in other RNAP structures, are not available for DNA contacts as they interact with DriD DNABD residues Glu128, Glu21, Glu27 and the backbone of Gly18 (Figure 3c)^63–65^. Hence, the αCTD-DriD contacts appear to anchor RNAP onto the promoter rather than enable promoter DNA binding by the αCTD.

### Test of DriD-ssDNA-RNAP-σ73-*CCNA_03891/CCNA_01149* structural model

The DriD-ssDNA-RNAP-σ73-*CCNA* structure indicates that DriD residues Gly18 and Ala20 are important for DriD interactions with both the RNAP β subunit and the RNAP αCTD. As the small sizes of the residues are key for these interactions and because they are juxtaposed next to a highly positive region of the αCTD, we generated a DriD(G18K-A20K) double mutant to assess effects on transcription. We first assayed the ability of this mutant to bind DriD target DNA in the presence of ssDNA. These data showed that the DriD(G18K-A20K) mutant bound DNA with essentially the same affinity (9.2 ± 2.0 nM) as WT (7.1 ± 1.8 nM) (Supplementary Figure 5). Having shown that the DriD(G18K-A20K) mutation does not affect target DNA binding, we next assessed the ability of the mutant to activate transcription in reporter assays. In these assays, while WT DriD was able to activate transcription from a reporter utilizing a *didA* reporter strain, the DriD(G18K-A20K) mutant showed no activation, thus supporting the structural model (Supplementary Figure 6). While the DriD-β and DriD-αCTD interfaces provide anchoring contacts for the RNAP, they are smaller than found in physiologically stable oligomers. Hence, the contacts between DriD and RNAP help anchor the polymerase on the promoter, but at the same time would not trap the polymerase onto the DNA, which would prevent promoter escape.

### The DriD 3HB is a trigger domain

A key finding from the DriD-ssDNA-RNAP-σ73-promoter structure is that the asymmetric binding mode of DriD on the DNA positions its 3HBs to make contacts with RNAP. HDX-MS and biochemical assays demonstrated that the DriD 3HB domains form autoinhibitory contacts with the DNABDs in the apo form and that this interaction prevents the latter’s contact with target DNA^51^. The data showed that ssDNA binding disrupts the DNABD-3HB interaction, allowing target DNA binding by the DriD DNABDs. Our new cryo-EM data indicate that the contacts to RNAP made by the DriD 3HBs would also be blocked in the apo state, i.e. in the absence of ssDNA. This indicates that removal of the autoinhibitory interactions is not only necessary to permit the DriD DNABD contacts with target DNA but also to allow interactions with RNAP.

To further probe the importance of the 3HB domain in RNAP binding, we performed binding assays. The structure indicates that DriD 3HB residues Arg121, Arg122, Pro125 and Glu128 are key for binding the αCTD. Hence, we used a DriD(R121E-R122E-P125E-E128R) mutant, previously shown to bind target DNA^51^, and analyzed its ability to bind the αCTD compared to WT DriD using fluorescence polarization (FP). In these experiments we first bound WT DriD or mutant DriD in the presence of ssDNA to fluoresceinated target DNA and then titrated in increasing concentrations of αCTD (α residues 161–248) (Methods). These experiments revealed that WT DriD-ssDNA-target DNA bound the αCTD with an apparent K_d_ of 30 ±5 μM, which, as would be predicted, is not a high affinity interaction that would trap the complex (Supplementary Figure 7). By contrast, no binding to the αCTD was observed by DriD(R121E-R122E-P125E-E128R) mutant in complex with ssDNA and target DNA (Supplementary Figure 7; Figure 3c). These data support the cryo-EM structure and indicate that DriD 3HB domain functions as a trigger domain that is targeted by allostery to switch between apo and RNAP bound states.

### Structure of the DriD-ssDNA-RNAPσ73 complexes with *didA* and *bapE* promoters

**Figure 4.**
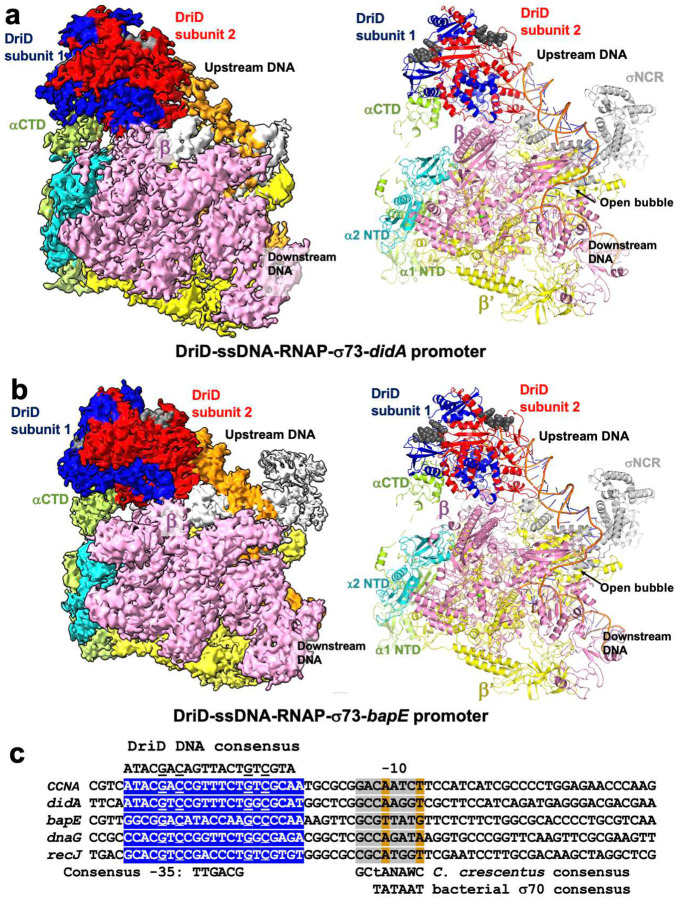
DriD-ssDNA-RNAP-σ73-*didA* and DriD-ssDNA-RNAP-σ73-*bapE* promoter complex structures. (**a**) Left, cryo-EM map of the DriD-ssDNA-RNAP-σ73-*didA* complex. Right, cartoon diagram of DriD-ssDNA-RNAP-σ73-*didA* complex. (**b**) Left, cryo-EM map of the DriD-ssDNA-RNAP-σ73-*bapE* complex. Right, cartoon diagram of DriD-ssDNA-RNAP-σ73-*bapE* complex. (**c**) Alignment of the promoter sequences utilized in the cryo-EM structures as well as *dnaG* and *recJ* showing a positionally conserved DriD DNA target site overlapping the −35 element and the same distance from the −10 element. The *C. crescentus* −35 hexamer is indicated as are the *C. crescentus* −10 consensus and the *E. coli* −10 consensus.

DriD was originally discovered as a regulator that activated transcription of the *didA* gene, which encodes the cell cycle inhibitor behind the *C. crescentus* non-SOS DNA damage response^36^. In addition to the *didA* promoter, DriD target DNA sites were identified in several additional promoters including *bapE*^37^. To understand DriD-mediated transcription activation of *didA* and *bapE* we next obtained structures of DriD-ssDNA-RNAP-*didA* and DriD-ssDNA-RNAP-*bapE* promoter complexes to resolutions of 3.98 Å and 4.24 Å, respectively (Supplementary Figures 8–12; Figure 4a–b). Examination of the promoters of these genes revealed that there is a clear DriD target DNA binding site in the *didA* promoter located close to or overlapping a putative −35 element^36^. However, the locations of the −35 and −10 elements in the *bapE* promoter are not evident and there are two putative DriD binding sites within this promoter (Supplementary Figure 1).

The structures of the DriD-ssDNA-RNAP-σ73-*didA* and DriD-ssDNA-RNAP-σ73-*bapE* promoter complexes reveal that DriD binds at the same position on these promoters as in the *CCNA_3891/CCNA_01149* complex (Figure 4a–b). Indeed, superimposition of the structures reveals that they adopt the same overall structures (Supplementary Figure 13). Hence, DriD binds the expected target site in the *didA* promoter, which overlaps the binding site for σ4 and the same relative position within the *bapE* promoter, which in both promoters appears to overlap the −35 promoter element.

### DriD activates transcription from promoters with nonoptimal promoter sequences

The *CCNA_03891/CCNA_01149, bapE* and *didA* promoters all have poor matches to the optimal −35 and −10 *C. crescentus* σ73 consensus sequences, which were revealed in studies in the Ely lab^66–68^. These analyses showed that although the *C. crescentus* RNAP-σ73 holoenzyme can recognize promoter elements from an *E. coli* σ70 promoter (i.e. a −10 element of T_−12_A_−11_T_−10_A_−9_A_8_T_−7_ and −35 of T_−35_T_−34_G_−33_A_−32_C_−31_A_−30_), it preferentially recognizes promoters with a longer −10 sequence, G_−14_C_−13_T_−12_A_−11_N_−10_A_−9_W_−8_C-_7_ (where W is A/T; N is A/T/G/C) and a −35 motif with the consensus, T_−35_T_−34_G_−33_A_−32_C_−31_G_−30_ (67–68). The longer −10 element in *C. crescentus* can be considered a combination of two elements, an extended −10 motif and −10 element. The extended −10 element encompasses −15 to −13 and has the consensus, T_−15_G_−14_N_−13_ (69–70). The positions of the bound σ2 and σ4 domains in our DriD-ssDNA-RNAP-σ73-promoter structures, allowed for the delineation of the −10 and −35 elements in the promoters. Notably, sequence alignments of the *didA* and *bapE* promoters with the *CCNA_03891/CCNA_01149* promoter revealed that the DriD binding sites in these promoters are all located the same distance from the −10 element in these promoters (Figure 4c). These analyses reveal that G_−14_ is among the few conserved nucleotides in the −10 motifs in these promoters. Notably, our structures show that the G_−14_ base positioned to be contacted by σ2 domain residue Arg481 and not σ3 domain residues as has been observed in other structures with extended −10 promoter motifs^69–70^. Based on these data and our structures, we the aligned the remaining DriD regulated promoters, *dnaG* and *recJ*, with the *didA*, *CCNA_03891/CCNA_01149* and *bapE* promoters. These alignments reveal clear DriD DNA-binding sites located the same distance from weakly conserved, yet recognizable *C. crescentus* −10 elements in these promoters (Figure 4c).

**Figure 5.**
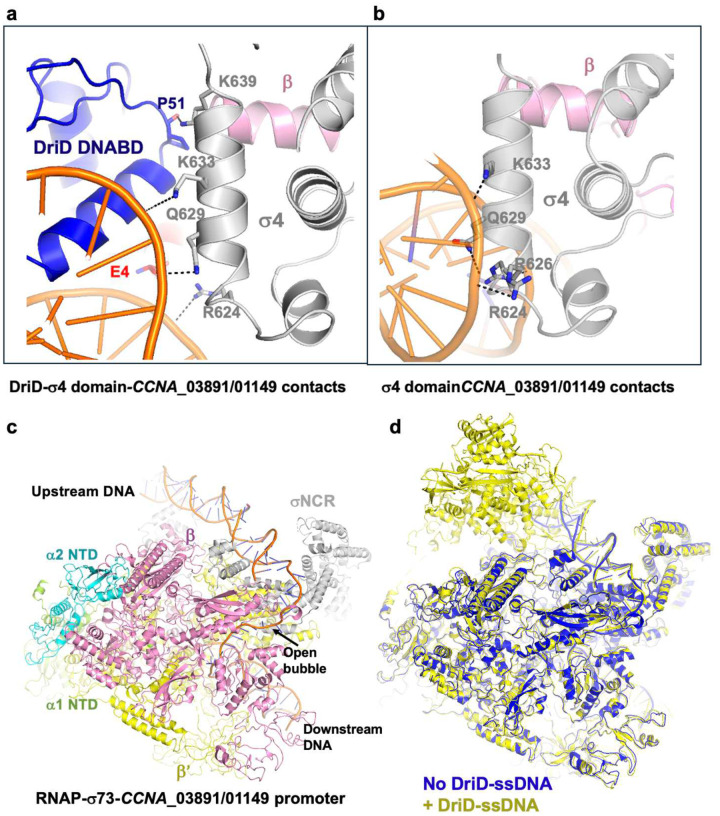
Structure of RNAP-σ73-*CCNA_03891/CCNA_01149* promoter complex and contacts to the −35 hexamer. (**a**) Close up of the DNA interactions by the σ4 subunit recognition helix in the DriD-ssDNA-RNAP-σ73-*CCNA_03891/CCNA_01149* promoter complex. Labeled and shown as sticks are residues that contact the DNA. (**b**) Close up of the DNA interactions by the σ4 subunit recognition helix in the and RNAP-σ73-*CCNA_03891/CCNA_01149* promoter complex. Labeled and shown as sticks are residues. **(c)** Cartoon structure of RNAP-σ73-*CCNA_03891/CCNA_01149* promoter complex with subunits labeled. (**d**) Overlay of the RNAP-σ73-*CCNA_03891/CCNA_01149* promoter complex (blue) and DriD-ssDNA-RNAP-σ73-*CCNA_03891/CCNA_01149* promoter complex (yellow).

The position of the σ4 domain on our structures also demarcates the position of the −35 motifs in these promoters and shows that the DriD DNABDs bind on either side of the −35 element; hence the DriD DNABDs sandwich the σ4 domain (Figure 5a). This is sterically possible because the σ4 domain binds a DNA face opposite the DriD DNABDs. That the DriD binding sites overlap the −35 sites is further bolstered by overlays with other RNAP complexes that place the −35 element and bound σ4 domain at the center of the DriD target site (Supplementary Figure 14). The −35 elements of the DriD bound promoters have even fewer matches to the *C. crescentus* −35 consensus hexamer than the −10 motif (the *CCNA_03891/CCNA_01149, didA, bapE, dnaG* and *recJ* −35 promoter elements have only 2, 2, 1, 1 and 1 match (out of 6)). Consistent with the poor −35 matches to the consensus, in the DriD bound structures the σ4 domains make few interactions to this motif (from Gln629, Lys633 and Arg624) and no base contacts (Figure 5a). This is in contrast to the contacts observed in structures of the σ4 domain bound to an optimal −35 motif, which shows multiple phosphate contacts as well as 5 base contacts^66^.

A 3.54 Å reconstruction of the RNAP-σ73 on the *CCNA_03891/CCNA_01149* promoter complex in the absence of DriD was obtained in our *CCNA_03891/CCNA_01149* promoter data and also showed few contacts to the −35 element by the σ4 domain (Figure 5b–d; Supplementary Figure 2). Hence, DriD binding to the promoter does not prevent specific contacts between the σ4 domain and the −35 element. Indeed, similar to the DriD bound structure, the complex without DriD reveals few contacts from the σ4 domain. Phosphate contacts are observed from Arg624 and Gln629 and possible long range electrostatic interactions are observed from Lys633 and Arg626 (Figure 5b). This complex solved in the absence of DriD was likely captured due to the high concentrations of the components in the sample. However, it should be noted that there is activity from this promoter in the absence of DriD, but the inclusion of DriD significantly enhances activity. Thus, our combined structural and biochemical data indicate that DriD activates transcription from promoters that have nonoptimal promoter elements by making several contacts to RNAP, helping to anchor it to these promoters.

## Discussion

The SOS response has been considered to be the major DNA damage pathway in bacteria. However, recent data has unveiled other DNA damage responses in bacteria^27–36^. The *C. crescentus* DNA damage response pathway mediated by DriD was one of the first non-SOS pathways described^41,43^. Yet, how DriD activates transcription has remained unknown. DriD is a WYL-domain containing protein, which is a family of proteins primarily found in bacteria that are identified by the presence of a Trp-Tyr-Leu motif. WYL proteins were originally characterized in connection to CRISPR (clustered regularly interspaced short palindromic repeats)-Cas (CRISPR-associated protein) adaptive immunity systems that are associated with certain CRISPR loci^41,71^. Subsequent bioinformatic studies revealed that WYL proteins are ubiquitous in bacteria, with >15,000 being identified. Most WYL proteins are categorized into class A (>10,000 proteins), which contain putative wHTH domains at their N-terminus^41^. Consistent with this, class A WYL proteins that have been characterized, including DriD, have been shown to function as transcription factors. These include regulators that are important for virulence in pathogenic bacteria^41^.

Early predictions suggested the WYL-domain exhibits a Sm-like structure, which is a fold utilized in nucleic acid binding^41^. Indeed, our initial studies of DriD captured it in complex with ssDNA and subsequent analyses confirmed that ssDNA is the DNA damage signal sensed by DriD and that it binds within its WYL domain^37^. This interaction was then demonstrated to stimulate the ability of DriD to bind target DNA sites and activate transcription of several promoters^37,38^. ChIP analyses revealed DriD binding sites in multiple promoters, with putative DriD target sites located proximal to the −10 element, the −35 element or in unknown relative locations to these elements in promoters where the motifs were not clearly identifiable^37^. Recent structures of transcription activation complexes have started to shed light on mechanisms utilized by transcription activators. However, DriD appears distinct from activator families that have been structurally analyzed based on a recent survey of bacterial transcription activator complex structures. This survey led to four major categorizations of transcription activators based on the location of the activator binding site within the promoter^52,72–87^. According to this classification, Type I activators bind upstream of the −35 element, type II bind downstream of the −35 box element, type III activate transcription without DNA binding and type IV bind both upstream and downstream of the −35 element. Type I activators include the Catabolite activator protein (CAP), AraC/XylS family proteins and Zur, a Fur family member^72–74^. These activators bind sites located between −41 to −81 on regulated promoters and primarily contact one or both of the αCTDs or the σ4 domain to anchor RNAP to the promoter. Type II activators also include CAP, which can bind and activate from multiple promoter locations, and MerR family regulators^75–78^. In type II promoter CAP-activated sites, CAP interacts with regions downstream of the −35 element and interfaces with the σ4 domain and/or the αCTD^75^. MerR-regulated promoters harbor nonoptimal spacings between these DNA motifs. By binding and distorting this region, MerR proteins align the −10 and −35 elements for optimal binding of the σ2 and σ4 domains, respectively^76–79^. The *C. crescentus* GcrA protein represents another type II activator. The structure of GcrA bound to RNAP and the *csM* promoter revealed that monomeric GcrA binds DNA downstream of the −35 box and interacts with the σ2 domain^53^. Type III activators include *E. coli* and *Francisella tularensis* SspA and *F. tularensis* SspA-MglA^80–81^. These proteins do not bind DNA and instead primarily contact the RNAP-holoenzyme σ factor. Finally, the Type IV activators, which bind up- and downstream of the −35 element, include the actinobacterial GlnR proteins and the λ CII transcriptional activator^82–84^.

The transcription activator that is the most similar structurally to DriD is the mycobacterial WYL-protein, PafB, which has been categorized as a type II transcription activator^86^. PafBC proteins also contain wHTH DNABDs, WYL and WCX motifs and regulate genes involved in DNA damage responses^51,85–86^. However, PafBC differs from the homodimeric DriD in that it is a heterodimer comprised of distinct subunits, PafB and PafC. Like DriD, PafBC is activated by binding ssDNA, however unlike DriD, the PafBC complex cannot bind its DNA target site in the absence of RNAP holoenzyme. The recent structure of FL PafBC in complex with ssDNA and RNAP revealed that only the PafB DNABD interacts with promoter DNA, at a site located −24 relative to the transcription start site, while residues from the PafC HTH subunit interact with the σ4 domain, By contrast, DriD can bind its target DNA independently of RNAP-σ73 and our cryo-EM structures show that the main contacts from DriD are to the β and αCTD of RNAP, not σ^84–86^. Thus, DriD employs a distinct activation mechanism from PafBC.

**Figure 6.**
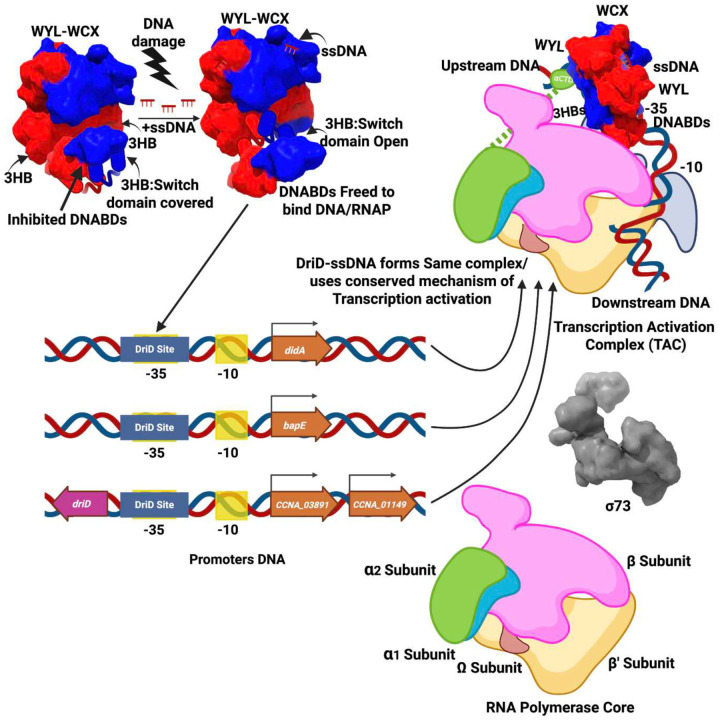
Schematic showing molecular mechanism of DriD regulated transcription. During non-DNA damage conditions, the apo form of DriD is present. Its 3HB regions interact with the DNABDs, inhibiting the latter’s contact with DNA. DNA damage leads to the production of ssDNA, which then binds the DriD WYL-domains, triggering allosteric conformational changes that leads to removal of the DNABDs from the 3HBs. This not only allows target DNA binding by the DNABDs but also frees the DriD 3HBs to interact with regions of the RNAP αCTD and β subunit when docked on DNA sites overlapping the −35 promoter element. In this way, DriD helps anchors RNAP-σ73 assemble suboptimal promoters.

Initial studies on DriD identified target DNA sites that were located at apparently distinct positions on different promoters, suggesting that DriD may function via multiple activating mechanisms, vis-a-vis CAP^72,75^. However, our structures of DriD bound to three promoters revealed that DriD employs a conserved activation mechanism that is different from the type I to IV categories^52^ as DriD interacts with DNA sites that directly overlap the −35 element. A characteristic feature of DriD activation is the asymmetric orientation of the DriD core region relative to its DNABD, which it adopts upon DNA binding. This conformation allows DriD to interface with multiple regions of the RNAP holoenzyme. In these interactions, DriD primarily employs it 3HB regions to interact with RNAP elements. The DriD 3HB has been shown to be the key to its allosteric mechanism whereby in the apo state of DriD, the 3HBs bind and sequester the DNABDs preventing their contacts with target DNA. ssDNA binding liberates the DNABDs for target DNA binding and it also frees the 3HBs, permitting their contacts with RNAP (Figure 6). Hence, our data reveals the 3HB as the nexus of DriD’s signaling and transcription activation functions.

An unanticipated finding from our studies is that DriD does not bind near the −10 element on the *driD* gene to impact transcription of its own gene. Instead, the DriD-ssDNA-RNAP-σ73-*driD* promoter complex revealed DriD and RNAP-σ73 oriented in the opposite direction on the promoter, with DriD binding a DNA site that overlaps the −35 element. Indeed, our analyses uncovered an operon, *CCNA_03891/CCNA_01149*, encoded in the opposite direction relative to *driD*, confirming that the promoter is bidirectional. Bidirectional promoters are defined as two genes with <1 kb between their transcription start sites^59^ and a bidirectional gene pair comprises two adjacent genes or operons whose transcription start sites are directed away from each other. Transcription at these promoters initiates from separate core promoters. That DriD regulates the *CCNA_03891/CCNA01149* operon was confirmed by our studies showing that DriD does not regulate its own expression but significantly activates transcription of the *CCNA_03891/CCNA_01149* operon.

Bidirectional promoters have been identified in multiple bacteria. In *C. crescentus*, bidirectional promoters include the promoter regulating the major chemotaxis operon and the chemotaxis associated gene A, *cagA*, and the promoter driving expression of the *ilvR* LysR-type regulator and in the opposite direction, *ilvD*, the gene encoding a branched chain amino acid biosynthesis enzyme^67,88^. In *E. coli* a recent study by Warman *et al*. found that 19% of genes are associated with a bidirectional promoter^88^. Bidirectional promoters, however, have been best studied in eukaryotes^41^. A detailed analysis of bidirectional promoters in humans revealed that ~11% of genes are transcribed from a bidirectional promoter^89^. The bidirectional arrangement in mammalian genes appears to be conserved; as several of these promoters in humans are also found in mice^89^. Interestingly, one of the main types of genes transcribed by bidirectional promoters (33.3% of bidirectional promoters) in humans are DNA repair genes^89^. It will be of interest to investigate whether bidirectional promoters in *C. crescentus* and other bacteria also regulate genes with related biological functions.

How transcription firing and/or coordination of transcription occurs from the *driD* and the *CCNA_03891/CCNA_01149* genes is currently unclear. Even in eukaryotes, where bidirectional promoters have been studied in detail, the regulatory function(s) of these promoters are poorly understood. Presumably, however when DriD is positioned to activate transcription of the *CCNA_03891/CCNA_01149* genes, as shown in our structure, it would be predicted to impede its own expression. Yet at the same time, the recruitment of the RNAP-σ73 holoenzyme to the promoter could enhance chances of DriD expression under certain conditions, such as when DriD dissociates and RNAP-σ73 is localized at the promoter. Moreover, promoter regions undergoing transcription develop negative supercoiling that combined with promoter melting could facilitate transcription from the oppositely oriented genes. Interestingly, the newly discovered operon (*CCNA_03891/CCNA_01149*) regulated by DriD encodes proteins with potential connections to DNA damage responses, as while the *CCNA_03891* gene has no known function, *CCNA_01149* encodes a TonB dependent transporter. TonB dependent transporters are associated with iron influx and studies have revealed that iron deficiency in *C. crescentus* leads to oxidative stress and subsequent activation of the SOS response^90^. Hence, transcription of this transporter could increase iron accumulation in cells in response to DNA damage or stress, via DriD activation. Combined with our previous analyses revealing that DriD regulates transcription of *recA*, these data suggest the intriguing possibility that there is cross talk between the SOS and DriD DNA damage response pathways.

In conclusion, our cryo-EM structures of DriD-ssDNA-RNAP promoter complexes have revealed unexpected layers in our understanding of transcription activation by this activator. This includes the clear delineation of the DriD binding and promoter elements within regulated promoters as well as the discovery of a *C. crescentus* bidirectional promoter. Indeed, these studies highlight the power of structural studies in revealing key promoter details as well as revealing the molecular mechanism of transcription activation by this regulator. The combined structures reveal that DriD stabilizes the RNAP holoenzyme on promoters that have nonoptimal RNAP binding elements (Figure 6). It does so using a unique mode of transcription activation involving the utilization of an asymmetric mode of DriD docking and a trigger domain mechanism that senses ssDNA produced during DNA damage.

## Methods

### Expression and purification of *C. crescentus* DriD and *C. crescentus* σ73

The genes encoding DriD, DriD(R121E-R122E-P125E-E128R), DriD(G18K-A20K), *C. crescentus* α subunit CTD (α residues 161–248) and *C. crescentus* σ73 were purchased from Genscript Corporation as genes codon optimized for *E. coli* expression (Piscataway, NJ, USA:http://www.genscript.com) and subcloned into pET15b such that a His-tag was expressed at the N-terminus of the proteins for purification. *E. coli* C41(DE3) cells were transformed with the vectors. Cells with each expression vector were grown at 37 °C in LB with 0.1 mg/mL ampicillin to an OD_600_ of 0.6, then induced with 0.5 mM isopropyl β-d-thiogalactopyranoside (IPTG) at 15 °C overnight. Cells were harvested by centrifugation (4,000 rpm, 4 °C, 15 min) and resuspended in buffer A (25 mM Tris-HCl pH 7.5, 300 mM NaCl, 5% (v/v) glycerol, 1 mM β-mercaptoethanol (βME)), then 1X protease inhibitor cocktail and 0.1 mg/mL DNase I were added. The resuspended cells were lysed by sonication and cell debris was removed by centrifugation (15,000 rpm, 4°C, 45 min). The supernatants were loaded onto a cobalt NTA column prepared in buffer A. For DriD, the column was washed overnight with 25 mM Tris-HCl pH 7.5, 800 mM NaCl, 2 M Urea, 13 mM imidazole, 5% (v/v) glycerol, and 1 mM βME. The next day, the column was washed with 300 mL of buffer A. For σ73, the column was washed overnight with 25 mM Tris-HCl pH 7.5, 800 mM NaCl, 10 mM imidazole, 5% (v/v) glycerol, and 1 mM βME. The proteins were eluted in steps of increasing imidazole concentration (20 mM, 30 mM, 50 mM, 100 mM, 200 mM, 500 mM, 1M, and 2M imidazole) in buffer A. Fractions were analyzed by SDS-PAGE and those containing the proteins were combined and concentrated in centricons and then buffer exchanged into low salt buffer A, 25 mM Tris-HCl pH 7.5, 150 mM NaCl, 5% (v/v) glycerol and 1 mM βME.

### Expression and purification of *C. crescentus* RNAP holoenzyme

For purification of *C. crescentus* RNAP core, a *C. crescentus* strain encoding a His-tagged ß’ subunit (*rpoC::rpoC-his10(kanR)*) of RNAP was grown in 25 mL of PYE media (0.2% Bacto Peptone, 0.1% yeast extract, 1 mM MgSO_4_ and 0.5 mM CaCl_2_) and agitated at 220 rpm at 30 °C for ~48 h. When cells reached an OD_600_ greater than 1.5, the cells were inoculated into flasks containing 1.5 L of PYE and grown overnight. The next morning, when the OD_600_ reached 0.6, the cells were spun at 5000 rpm, 4 °C for 20 min. Cell pellets were separated from the supernatant to avoid the pellets from dissolving back into the supernatant. The pellets were resuspended in 30 mL of lysis buffer P (50 mM Tris-HCl pH 8, 300 mM NaCl, 1 mM βME, and 5% (v/v) glycerol) then 1X protease and 10 μL of 10 mg/mL DNAse I were added per 100 mL of lysate. Cells were lysed by sonication 8 times for 30 s in 40 mL aliquots before 3 cell disruption steps using a Microfluidizer were carried out. The lysate was spun at 16,000 rpm for 45 min at 4 °C. A Ni-NTA column was prepared in lysis buffer P and the supernatant was applied to the column and washed with 300 mL of buffer P plus 10 mM imidazole. The RNAP core complex was eluted in steps, using 30 mL of buffer P plus 20 mM, 40 mM, 60 mM, 80 mM, 100 mM, 250 mM, 500 mM imidazole. Protein purity was assessed by analysis on SDS gels. The purified RNAP was then buffer exchanged 3X into a buffer composed of 12.5 mM Tris-HCl pH 8, 100 μM EDTA, 100 μM DTT, 150 mM NaCl. To obtain the RNAP holoenzyme (i.e the RNAP-σ73 complex), the purified *C. crescentus* σ73 was mixed with *C. crescentus* RNAP at a ratio of 5-fold molar excess σ73, then incubated overnight at 4 °C on a rotator shaker. the RNAP-σ73 holoenzyme was further purified by size exclusion chromatography on a Superdex S-200 column. Fractions containing the RNAP holoenzyme were combined and buffer exchanged 3X into cryo-EM buffer (20 mM Tris-HCl pH 8, 5 mM MgCl_2_, 100 mM NaCl, 1 mM βME).

### Reconstitution of DriD-ssDNA-RNAP holoenzyme-promoter DNA complexes

To generate the DriD-ssDNA-RNAP-promoter complexes, 3 μM RNAP holoenzyme, 3 μM DriD, 1.33 μM 7mer ssDNA (5′-TGTCTAT-3′), and 3 μM promoter DNA obtained from IDT (below) were combined and incubated at rt for 30 min. Complexes were all prepared in cryo-EM buffer. *didA* promoter sequence: 5’-GGGCGCAGCGCGGCAAGTTCAATACGTCCGTTTCTGGCGCATGGCTCGGCCAAGGTCGCTTCCATCAGATGAGGGACGACGACATGACCGACTTCGCCA-3’. *driD* (*CCNA_03891/CCNA_01149*) promoter sequence: 5’-AGGCGAAGCGGGTCATCGAAGGTCCTTGGGTTCTCCAGGGGCGATGATGGAAGATTGTCCGCGCATTGCGACAGAAACGGTCGTATGACGGGCGGCATG-3’. *bapE* promoter sequence: 5’-GCGCAGCGTTGGCGGACATACCAAGCCCCAAAAGTTCGCGTTATGTTCTCTTCTGGCGCACCCCTGCGTCAAGCCGGGACGCCGCGACAT-3’.

### Cryo-EM grid preparation

For grid preparation, UltrAufoil R1.2/1.3 Au 300 (Quantifoil) holey gold grids were glow-discharged (PELCO easiGlow) for 300 s, and 3 μL of purified samples of each 3 reconstituted complexes of DriD-ssDNA-RNAP-σ73-complexes with *didA, CCNA_03891/CCNA_01149 and bapE* promoters were applied at rt at 90% humidity on separate grids. Following a 10 s incubation period, the grids were blotted for 1.5–2 s and plunged into liquid ethane using a Leica EM GP2 (Leica Microsystems). Vitrified grids were clipped with Autogrid sample carrier assemblies (Thermo Fisher) immediately before imaging.

### Cryo-EM data collection

Screening of the DriD-ssDNA-RNAP-σ73 promoter complex grids were performed on a Thermo Fisher Tundra Cryo-TEM operated at 100 kV and equipped with a Ceta Camera with dose fractionation and semi-automated sample loading. Grids deemed optimal for data collection were then loaded onto a Titan Krios Gi3 (Thermo Fisher at Duke University) electron microscope equipped with a BioQuantum Energy Filter (Gatan) and data were collected for each complex. For each data collection, the microscope was aligned using a cross-grating replica TEM grid; coma-free alignment and astigmatism corrections were done using the AutoCTF Sherpa functionality (Thermo Fisher software). Data were collected using Latitude S (Digital Micrograph (Gatan)) software running an image shift pattern at 0° stage tilt in counting mode with a nominal magnification of 81,000X/105,000X with a physical pixel size of 1.08/0.873 Å pixel and a defocus range of −0.8 to −2.5 μm. A 100 μm objective aperture and 20 eV energy filter were used for all data collection. A gain reference was collected in a Digital Micrograph (Gatan) before starting data collection and a new dark reference was collected every 2 h during data collection. Parameters for all datasets are listed in Supplementary Table 1.

### Cryo-EM data processing

Cryo-EM flow diagrams demonstrating data processing steps and final structure analyses are shown in Supplementary Figures 2, 8–9. Image processing of the complexes were done using cryoSPARC v4.2, 4.3, 4.4.1, 4.5.3, and 4.6.2 (Structural Biotechnology)^91^ and RELION-5-beta^54–56^ software and the spIsoNet software suit in the RELION-4 pipeline (used for misalignment correction, 3DFSC calculation and anisotropy correction). Gain corrected movie frames were aligned with the Patch Motion module in cryoSPARC and CTF estimations were done with Patch CTF. Low-quality micrographs with high relative ice thickness, high motion pixel distances, high motions curvature, higher defocus tilt angle or low CTF fit resolution were removed from each dataset by manual curation. The preprocessed selected movies of each dataset were used for particle picking using a blob picker and then template-based particle picking was performed. Low-resolution *ab-initio* models were generated with the initial sets of particles that went through one or two rounds of 2D classification. Iterative rounds of 2D classification, multiclass *ab-initio* reconstructions, and heterogeneous refinement further removed particles that did not contribute to high-resolution reconstructions of the complexes. Global consensus reconstructions of each DriD-ssDNA-RNAP-σ73-promoter-complex were then carried out using non-uniform refinement^92^ in cryoSPARC with C1 symmetry. The final set of particles were extracted with an unbinned box size of 384/450 pixels. These particles were exported using csparc2star.py^93^ from the pyem v0.5 script package and particles were imported into RELION-5-beta-0–2commit and subjected to iterative 3D classification with and without image alignment settings. A 320/450 Å mask diameter and reference map was used and the volume imported from CryoSPARC from non-uniform refinement^91^ and low pass filtered to 50–60 Å using blush regularizations^54–56^. The 3D classes and their respective statistics (Number of particles, percentage of particles in each class, and their respective resolution) for each complex are shown in the data processing flow diagrams in Supplementary Figures 2, 8–9. The classes representing the DriD-ssDNA (and non-DriD bound complex in the *CCNA* promoter) and were subjected to 3D auto-refinement, applying C1 symmetry in RELION-5 or cryoSPARC Non-Uniform refinements^92^. CTF refinements were done for each class of respective promoter complexes to correct for magnification anisotropy, fourth-order aberrations, per-particle defocus and per-particle astigmatism, followed by another 3D auto-refinement/Non-Uniform refinement.

The resultant maps and particles were then subjected to spIsoNet^94^ Misalignment Correction (MC) as integrated into the refinement pipeline of RELION-4^54–56^ using the ‘–external_reconstruct’ function in RELION_3D-auto-refine. The RELION_wrapper.py in spIsoNet^93^ was executed after each iteration of RELION_3D-auto-refine when the refinement reached a finer angular sampling. The two unfiltered half-maps generated from the MC module and a soft solvent mask were used as inputs to the spIsoNet’s Anisotropy Correction (AC) module in the conda environment of the spIsoNet software suit to calculate 3DFSC volume representing the anisotropic resolution for the reconstruction. Then, a ‘reconstruct’ step was performed to train a neural network to recover the missing information based on 3DFSC. The ‘reconstruct’ step, performed on the half-maps generated a neural network and anisotropy-corrected half-maps. The subsequent post-processing of the half-maps for sharpening and gold-standard FSC determination was performed in the RELION-4 software suit^54–56^.

The half maps generated through MC and/or AC were further postprocessed with density modification and soft masking outside the refinement mask using phenix.resolve_cryo_em^95^ in the Phenix suite. This was done for all maps except the *C. crescentus* RNAP-σ73-*CCNA_03891/CCNA_01149* promoter complex map, as density modification did not improve this map. The local resolution maps shown in Supplementary Figures 2, 8–9 were generated in ChimeraX^96^ using the density modified maps generated from density modification in the Phenix/RELION/CryoSPARC^91^. The atomic models were first fit as rigid bodies in ChimeraX1.7^97–98^ using the 7YE1 structure and DriD-ssDNA-target DNA complex (7TZV) as starting models for generating the RNAP-σ73-DriD-ssDNA-*CCNA_03891/CCNA_01149* model. Detailed fitting was done in Coot followed by Phenix real-space refinement^95,99^. The RNAP-σ73-DriD-ssDNA-*bapE* promoter and RNAP-σ73-DriD-ssDNA-*didA* promoter structures were fit using the RNAP-σ73-DriD-ssDNA-*CCNA_03891/CCNA_01149* structure as a starting model and refitting was done in Coot^99^. All refined coordinates were inspected and modified in Coot^96^, as needed and validated using Phenix^93^ and CCP-EM software suite^100^. In all but the RNAP-σ73-DriD-ssDNA-*didA* promoter structure, the σ73 NCR, β residues 980–1030 and β’ residues 947–1119 harbored weak density, but could still be modeled. In the RNAP-σ73-DriD-ssDNA-*didA* promoter structure the DriD dimer was, overall, well resolved in the 4.5 Å map. However, density for the σ73 NCR and most of β’ residues 947–1119 was absent from this map. Hence, we used a low-resolution map (7 Å map) generated during 3D classification which had density for these regions, to complete the fit.

### Fluorescence polarization (FP) studies

Fluorescence polarization studies were used to assess binding of the αCTD to WT DriD and DriD(R121E-R122E-P125E-E128R) in the presence of 100 μM ssDNA (5’-TGACTAT-3’) in a buffer consisting of 25 mM Tris-HCl pH 7.5, 150 mM NaCl, 2.5 % (v/v) glycerol. First the DriD proteins were bound to F-DNA (top strand: 5’-ATACGACAGTAACTGTCGTAT-3’) and then increasing concentrations of αCTD was titrated into the tube. A second binding event indicated CTD interaction with DriD. FP was also performed to analyze binding of the DriD(G18K-A20K) mutant to the F-DriD target DNA in the presence of 100 μM ssDNA (5’-TGACTAT-3’) and compared to WT binding. Three technical replicates were performed for each experiment. Normalized change in millipolarization (mP) was plotted against increasing protein concentration, and binding affinities were calculated by fitting the curves with GraphPad Prism. Change in mP units was normalized by (*A* – *A*_0_)/(*A*_max_−*A*_0_) where *A* is change in mP reading, *A*_0_ is the initial mP value before addition, and *A*_max_ is the maximal mP reading upon binding saturation. The error in *K*_d_ was determined as the standard deviation between the calculated *K*_d_s for three runs.

### Strain Construction

Plasmids and *Caulobacter crescentus* strains used in this study are listed in Supplementary Tables 2 and 3, respectively. DNA oligonucleotides used in strain construction are listed in Supplementary Table 4. Genomic DNA of *C. crescentus* CB15N was used as a template for PCR amplifications unless noted otherwise.

To generate pNPTS138-*hfab*::*lacZ*, approximately 600-bp flanking upstream and downstream homology regions (UHR and DHR) of the *hfaB* gene (*CCNA_02712*) were cloned sequentially into pNPTS138: The UHR of *CCNA_02712* was amplified by PCR using primers oKRG627 and oKRG628 and restriction cloned into the AflII and SalI sites of pNPTS138. The DHR of *CCNA_02712* was then amplified by PCR using primers oKRG629 and oKRG630 and restriction cloned into the HindIII and SpeI sites of the resulting plasmid. Then, *lacZ* was PCR amplified with primers oKRG799 and oKRG800 and restriction cloned into the SalI and HindIII sites of the resulting plasmid, creating pNPTS138-*hfaB*::*lacZ*.

To generate the plasmids pNPTS138- *hfab*::P_*didA*_-*lacZ*, pNPTS138- *hfab*::P_*driD*_-*lacZ* and pNPTS138- *hfab*::P_*03891/01149*_-*lacZ*, the promoter region of *didA*, *driD* and *CCNA_03891/01149* were amplified via PCR using primers oKRG783/oKRG784, oKRG785/oKRG786 and oKRG799/oKRG800, respectively. The PCR product were then purified, digested with SalI and BamHI, and ligated into the double-enzyme cut corresponding pNPTS138-*hfab*::*lacZ* plasmid. Five microliters of ligation was then transformed into chemically competent *E. coli* DH5α cells and correct clones were identified.

To generate the plasmid pRVMCS-2::P_*driD*_-*driD* (G18K A20K), the region from the *driD* promoter up to 15 bp after the stop codon of *driD* was amplified in two parts, using oKRG317 paired with either oKRG798 and oKRG316 paired with either oKRG797. The two amplicons of *driD* were combined with fusion PCR with the outside primers oKRG316 and oKRG317. The fused product was gel-purified, digested with SacI/SacII and ligated into SacI/SacII-digested pRVMCS-2.

To generate the reporter strains KG720, KG724 and KG728, 1.5 μL of the plasmids pNPTS138-*hfab*::P_*didA*_-*lacZ*, pNPTS138-*hfab*::P_*03891/01149*_-*lacZ* and pNPTS138-*hfab*::P_*driD*_-*lacZ* were electroporated into 50 μL of competent WT CB15N cells (ML76). The entire sample was plated on PYE agar plates containing kanamycin, and surviving colonies were inoculated into PYE media for 18 hours. 5 μL of the culture was plated with 45 μL of plain PYE media onto PYE and 3% sucrose plates. The resulting colonies were plated on both PYE/Kanamycin plates and plain PYE plates, and those that maintained the sensitivity to Kan were PCR-verified. As above, the reporter strains KG721, KG725 and KG729 were generated via electroporation of pNPTS138- *hfab*::P_*didA*_-*lacZ*, pNPTS138- *hfab*::P_*03891/01149*_-*lacZ* and pNPTS138- *hfab*::P_*driD*_-*lacZ* into ML2174, respectively.

To generate strains KG722, KG726 and KG730, the empty vector MTLS4424 was electroporated into competent cells of KG721, KG725, and KG729, respectively. To generate strains KG723, KG727 and KG731, the plasmid pRVMCS-2::P_*driD*_-*driD* was electroporated into competent cells of KG721, KG725, and KG729, respectively. To generate strain KG732, the plasmid pRVMCS-2::P_*driD*_-*driD* (G18K A20K) was electroporated into competent cells of KG570, respectively.

PCR was performed with Phusion HF DNA polymerase with 5 × Phusion GC reaction buffer (NEB). Each reaction contained 10 μL of buffer, 4 μL of dNTPs (final concentration of 200 μM), 5 μL of a 10 μM forward and reverse primer mix, 50 ng of template, 10 μL of 3 M betaine monohydrate (Sigma), 1 μL of DMSO, 0.5 μL (1 unit) of polymerase, and nuclease-free water to 50 μL. Two-step cycling was performed as follows: an initial 30 s at 98°C, then 10 s at 98°C and 30 s/kb at 72°C for 34 cycles, and a final 5 min at 72°C. For fusion PCR, 50 ng of the largest fragment and equimolar of other fragments were added to the reaction, and an extra 60°C annealing step was added to the standard PCR cycling. Final PCR products were digested with the noted restriction enzymes and ligated into correspondingly digested plasmid. Five microliters of the ligation mixture were then transformed into chemically competent *E. coli* DH5α cells. All resulting plasmids were verified by nanopore sequencing by Plasmidsaurus.

### RNA Isolation

Relevant strains were grown in 150 mL flasks with 15 mL PYE until mid-exponential phase (OD600 = 0.2–0.3) followed by incubation with zeocin (15 μg/mL) for 45 min. Then, 500 mL of culture was centrifuged, and the pellets were frozen at −80°C. 400 mL of heated Trizol reagent was to the tubes and vortexed for 10 min at 65°C. The mixture was frozen again at 80°C for 10 min before the cells were thawed and pelleted again. The RNA was further purified using the protocol from the Zymo Direct-zol kit (Cat # R2051-A).

### Reverse transcription quantitative polymerase chain reaction (RT-qPCR)

A mixture of 2.5 mL of isolated RNA, 0.5 mL of random hexamer primers (100 ng/ml), 0.5 mL of a dNTP mix (10 mM each), and 3 mL of H_2_0 was heated to 65°C for 5 minutes (min) and then placed on ice for one min. Then, 2 mL of first strand buffer, 0.5 mL of DTT (100 mM), 0.5 uL of RNase inhibitor, and 0.5 mL of Superscript III (200 U/mL) RT were added to each tube. The mixtures were cycled through 25°C for 10 min, 50°C for 60 min, and 70°C for 15 min before the samples were incubated with 1 mL of RNase H at 37°C for 20 min. The cDNA samples were then diluted 10-fold. In each well, 5 mL of SYBR Green, 3.5 mL H2O, and 0.25 mL of each relevant primer was added to 1 mL of the diluted cDNA. The qPCR plate was run at 95°C for 5 min, and then 40 cycles of 95°C for 5 s, 60°C for 30 s, and 72°C for 20 s. Finally, a melting curve was set at 95°C for 5 s, 55°C for 1 min and 97°C continuously decreasing by 0.11°C/s.

### β-galactosidase Assays

To quantify the β-galactosidase activity, reporter strains were grown in biological triplicate to mid-log phase (OD600 = 0.2–0.3) and treated with 15 mg/mL of zeocin for 45 min. Then, 800 mL of each sample was pelleted and resuspended in Z-buffer containing BME (2.7 μL per mL). The harvested cells were then degraded with 100 mL of chloroform and 50 mL of 0.1% SDS. Following an incubation period of 15 min at 30°C, ortho-nitrophenyl-β-galactoside (4 mg/mL in Z-buffer + BME) was added, and the reaction was terminated by addition of 500 μL 1M Na_2_CO_3_ once each sample generated a yellow color. The assay and relevant activity calculations were done as previously described^101^.

## Supplementary Material

Supplementary Files

This is a list of supplementary files associated with this preprint. Click to download.

- SinghSupplementatyMatMicroJuly12.pdf
- SupplementaryMovieFiguresLegends.docx
- SupplementaryMovie1.mp4
- D1000297728valreportfullannotateP1.pdf
- pdbles.zip

## Data Availability

The cryo-EM structures generated in this study have been deposited in the Protein Data Bank under the codes 9PFV, 9PGA, and 9PFQ for the DriD-ssDNA-RNAP-σ73-complexes with the *CCNA_03891/CCNA_01149, didA*, and *bapE* promoters, respectively and 9PGH for the RNAP-σ73-*CCNA_03891/CCNA_01149* promoter structure. The corresponding cryo-EM maps have been deposited in the EMDB under the codes EMD-71615, EMD-71624, and EMD-71610 for the DriD-ssDNA-RNAP-σ73-complexes with the *CCNA_03891/CCNA_01149, didA*, and *bapE* promoters, respectively and EMD-71632 for the RNAP-σ73-*CCNA_03891/CCNA_01149* structure. Any additional information reported in the manuscript is available from the corresponding author upon request.
